# Dengue seroprevalence among asymptomatic blood donors during an epidemic outbreak in Central-West Brazil

**DOI:** 10.1371/journal.pone.0213793

**Published:** 2019-03-25

**Authors:** Svetoslav Nanev Slavov, Daiani Cristina Cilião-Alves, Filipe Almeida Carvalho Gonzaga, Drielly Rodrigues Moura, Ana Carolina Alves Melo de Moura, Lorena Aparecida Gonçalves de Noronha, Évelin Mota Cassemiro, Bárbara Maciel Sidou Pimentel, Fabiano José Queiroz Costa, Grasiela Araújo da Silva, Doralina do Amaral Rabello Ramos, Wildo Navegantes de Araújo, Simone Kashima, Rodrigo Haddad

**Affiliations:** 1 Blood Center of Ribeirão Preto, Faculty of Medicine of Ribeirão Preto, University of São Paulo, Ribeirão Preto, São Paulo, Brazil; 2 Department of Internal Medicine, Faculty of Medicine of Ribeirão Preto, University of São Paulo, Ribeirão Preto, São Paulo, Brazil; 3 Euro-American University Centre – UNIEURO, Brasilia, Federal District, Brazil; 4 Faculty of Ceilândia, University of Brasilia, Brasilia, Federal District, Brazil; 5 Center for Tropical Medicine, University of Brasilia, Brasilia, Federal District, Brazil; 6 Blood Center of Brasilia, Brasilia, Federal District, Brazil; 7 Central Public Health Laboratory of Federal District – LACEN-DF, Brasilia, Federal District, Brazil; 8 Laboratory of Molecular Pathology of Cancer, Faculty of Health Sciences and Medicine, University of Brasilia, Brasilia, Federal District, Brazil; 9 Department of Clinical, Toxicological and Bromatological Analyses, Faculty of Pharmaceutical Sciences of Ribeirão Preto, University of São Paulo, Ribeirão Preto, São Paulo, Brazil; CEA, FRANCE

## Abstract

Dengue virus (DENV) transmission by blood transfusion is an important route of viral acquisition during outbreaks. The prevalence of DENV markers (viral RNA, NS1, anti-DENV IgM, and IgG) among blood donors in Central-West Brazil has never been evaluated. Our aim was to evaluate the full set of serological and molecular markers for DENV among blood donors of the Federal District of Brazil during an extensive outbreak in 2016. We found an anti-DENV IgM prevalence of 6.74% (n = 32/475). Of 475, 20 samples (4.21%) were also anti-DENV IgG positive. All samples were non-reactive for NS1 and DENV RNA. Our results imply that a significant proportion of the tested donors had experienced asymptomatic infection. More studies are necessary to evaluate the real prevalence of DENV viremia in blood donors from the Federal District of Brazil and if specific measures are needed to routinely test the blood donors for DENV RNA during outbreaks.

## Introduction

Dengue viruses (DENV 1–4) are etiological agents of Dengue fever, which is the most important arboviral disease in the world [1]. DENV 1–4 are primarily transmitted by arthropod mosquito vectors of the *Aedes* genus (*A*. *aegypti* and *A*. *albopictus*). However, as indicated by extensive DENV outbreaks with a significant proportion of asymptomatic cases (up to 50%), this arboviral disease can also be transmitted by blood transfusion in the form of transfusion-transmitted DENV (TT-DENV) [2,3]. Transfusion-transmitted DENV cases have been documented in distinct, mainly tropical, geographic locations, including Central and South America and Southeast Asia, e.g., Brazil [3,4], Puerto Rico [5], Pakistan [6], Hong Kong [7], and Singapore [8].

Brazil is the country with the largest number of reported DENV cases annually in the Americas [3], and the magnitude of outbreaks and severity of the disease has been increasing, especially in recent years [9]. Moreover, there is active DENV transmission within most of the regions of the Brazilian territory nationwide, even in the absence of significant outbreaks, which can additionally complicate the surveillance of this arbovirosis [10]. Despite this scenario, Brazilian Health Authorities have never implemented any specific measures to prevent TT-DENV during outbreaks, in contrast to the measures undertaken to contain the TT of West Nile Virus in the USA [4].

We conducted a pilot study to evaluate the seroprevalence of anti-DENV IgM and the presence of viral RNA and NS1 positivity in a cohort of well-characterized blood donors during a large DENV outbreak in Brasilia, the Federal District of Brazil, in 2016.

## Material and methods

### Study period

**Fig 1:**
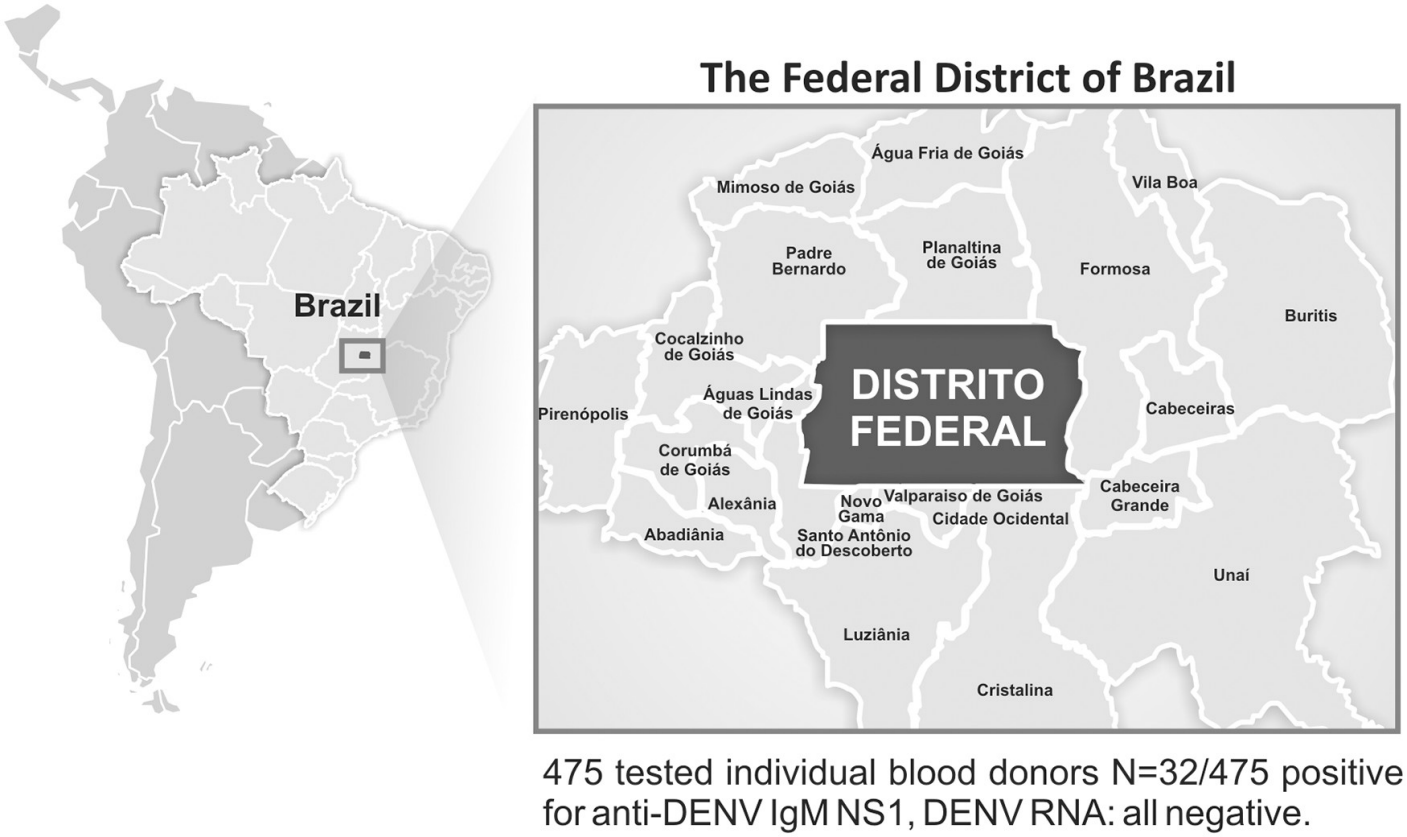
Geographic localization of the Brazilian Federal District. Geographic localization of the study and the key findings obtained in this study in blood donors.

**Fig 2:**
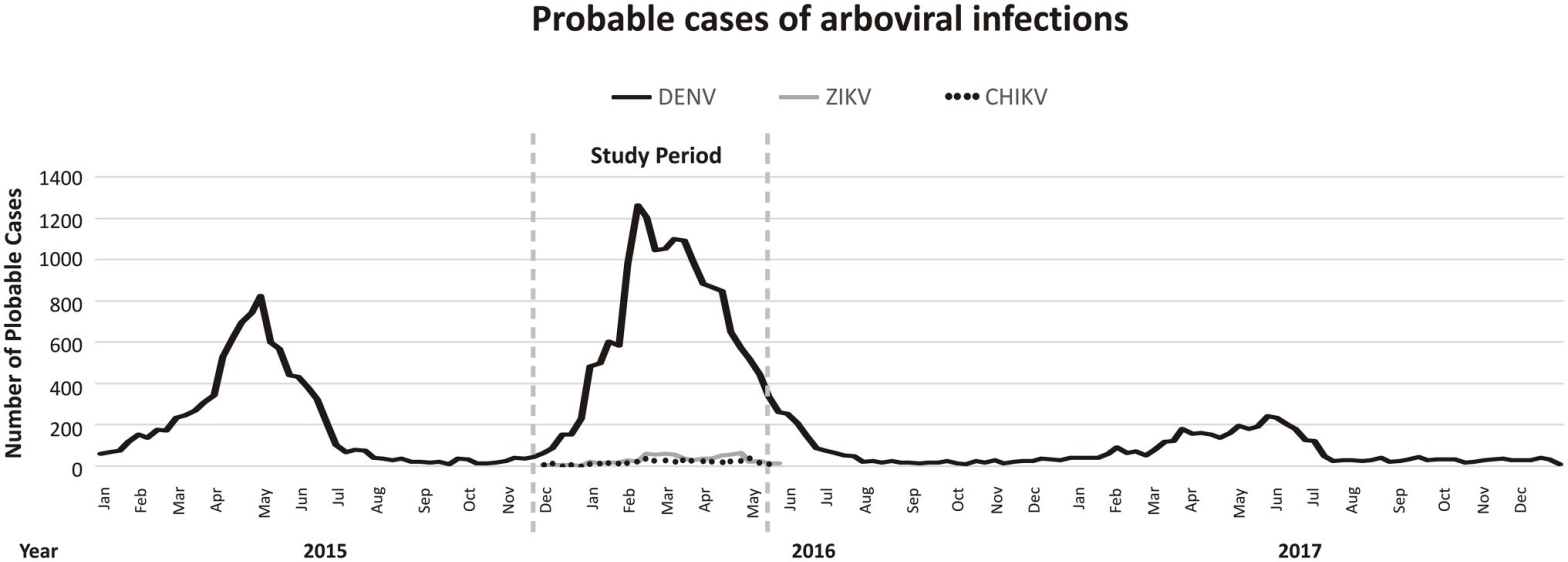
Notifications of probable cases of arboviral infections in the Federal District. The number of notifications was accessed in the Epidemiological Bulletin for Dengue, Chikungunya, and Zika of the Federal District, Brazil (primary source: Notification System, SINAN). Dashed lines determine the period in which samples were collected (DENV outbreak period).

Sample collection was performed between December 2015 and May 2016 in the Blood Center of Brasilia, Federal District, Brazil (Central Plateau, Central-West Brazil, 15°47´S 47°45´W) (Fig 1). In order to define the period of our study, we consulted the Epidemiological Bulletin for reported cases of Dengue, Chikungunya, and Zika for the Federal District of Brazil, which uses as a primary data source the Notification System (SINAN). The number of probable Dengue cases was obtained for the period between 2015 and 2017, and we identified the peak of probable DENV cases. The numbers of probable ZIKV and CHIKV cases were also obtained for the period of the study (Fig 2).

### Sample size calculation

**Fig 3:**
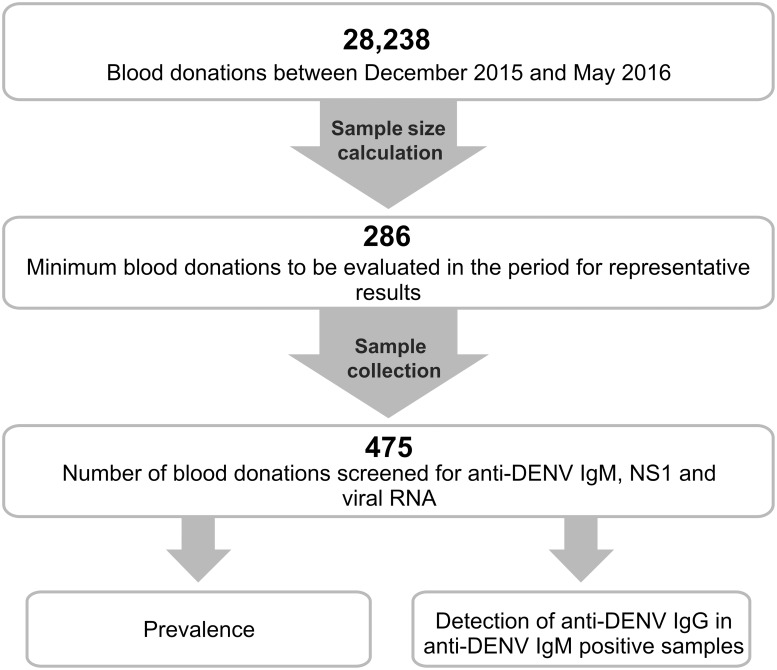
Experimental strategy for the study. Experimental strategy for DENV marker screening in a significant number of blood donations; samples positive for DENV markers were used for prevalence determination. Anti-DENV IgM-positive samples were also tested for Anti-DENV IgG to differentiate early primary infection from late primary infection or early secondary infection.

During the period of this study (December 2015 and May 2016), a total of 28,238 plasma samples were collected from volunteer blood donors who were eligible for blood donation. An online epidemiology calculator for test proportions was used (http://www.winepi.net/) [11] to calculate an appropriate sample size to be tested (Confidence Interval [CI]: 95%; desired precision ± 1%). We calculated that a minimum sample size of 286 was required (Fig 3). Considering that the rarest event in DENV infection in blood donors is the presence of viral RNA, for this calculation, we adopted a true estimated prevalence of 0.75%, based on a previous study performed on blood donors in the Pernambuco and Rio de Janeiro States, Northeast and Southeast Brazil, respectively [3].

### Sample collection

A total of 475 plasma samples were included to be tested in our study (Fig 3). The sample distribution by month was as follows: 82 samples from December 2015, 72 from January, 79 from February, 81 from March, 81 from April, and 80 from May 2016. Briefly, 5 mL of whole blood was obtained from each blood donor in sterile vacutainers with EDTA (Vacuette, Greiner Bio-One, Americana, São Paulo, Brazil) at the time of blood donation. The samples were processed by low-speed centrifugation (1931 × *g*, 10 min) to separate the plasma. If not used on the same day, the plasma was stored at −80 °C until use. The study was approved by the Research Ethics Committee of the Health Secretariat of the Federal District, Brazil (CEP/SES/DF, protocol N° 62718016.0.3001.5440).

As many arboviruses are endemic to Brazil and due to the introduction of ZIKV and CHIKV causing large outbreaks, blood donors are asked to respond to a questionnaire that includes queries on possible recent symptoms, including the presence of spots on the skin, fever, reddened eyes, pain in the body or joints, headache, swelling in the hands or feet, sore throat, coughing, and vomiting. In addition, volunteers are asked if they have ever had ZIKV or CHIKV infections based on clinical signs or have travelled to regions in which these infections are endemic. If positive responses are received, such donors are deferred from donating blood for 30 days. Also, candidates who have had sexual contact in the last 90 days with partners who have received a clinical and/or laboratory diagnosis of ZIKV and CHIKV are also considered unqualified to donate blood for 30 days. Finally, volunteers who have travelled to endemic regions with ongoing outbreaks of CHIKV are also considered unqualified for donation for 30 days.

### NS1 testing for DENV

All collected samples were tested for DENV NS1 in order to evaluate the presence of an early marker of DENV infection. For this purpose, the Platelia Dengue NS1 Ag (Bio-Rad Laboratories, Inc, Hercules, California, USA) was used following the manufacturer’s instructions. The results were expressed in terms of index value: if the index value was <0.5, the samples were considered non-reactive for DENV NS1; if the index value was between 0.5 and 1.0, the samples were considered equivocal for DENV NS1; and if the index value was 1.0 or more, the samples were considered reactive for DENV NS1.

### Serological testing for anti-DENV IgM

All collected samples were tested for anti-DENV IgM in order to evaluate the presence of recent infection. For this purpose, the Panbio Dengue IgM Capture ELISA (Abbott Laboratories, Chicago, Illinois, USA) was used following the manufacturer’s instructions. The results were expressed in PANBIO units, considering a result <9 as non-reactive, between 9 and 11 as in the gray zone, and >11 as reactive for anti-DENV IgM.

### Serological testing for anti-DENV IgG

Only anti-DENV IgM-positive samples were tested for anti-DENV IgG in order to evaluate infection dynamics. For this purpose, the Anti-Dengue Virus ELISA (IgG) (Euroimmun Medizinische Labordiagnostika AG, Lübeck, Germany) was used following the manufacturer’s instructions. The results were expressed in relative units (RU/mL): <0.8 RU/mL was considered negative, between 0.8 and 1.1 was considered within the gray zone, and >1.1 RU/mL was considered positive for anti-DENV IgG.

### Serological testing for anti-Zika virus (ZIKV) IgM

To rule out cross-reactivity to anti-ZIKV IgM, anti-DENV IgM-positive samples were tested for anti-ZIKV and anti-DENV IgM using MAC-ELISA protocols as described previously [12] with antigens provided by the Centers For Diseases Control and Prevention (CDC), Atlanta, GA, USA. In accordance with the protocol, the results were expressed as absorbance and compared with a standard curve. A result <0.180 was considered negative, between 0.180 and 0.220 was classified in the borderline zone, and >0.220 was considered positive for anti-ZIKV IgM.

### Serological testing for Anti-Chikungunya virus (CHIKV) IgM

To rule out cross-reactivity to CHIKV, all anti-DENV IgM-positive samples were tested for anti-CHIKV IgM. For this purpose, the Anti-Chikungunya virus ELISA (IgM) kit (Euroimmun Medizinische Labordiagnostika AG, Lübeck, Germany) was used following the manufacturer’s instructions. The results were expressed in relative units (RU/mL): a value <0.8 RU/mL was considered negative, between 0.8 and 1.1 RU/mL was considered in the borderline zone, and >1.1 RU/mL was considered positive for anti-CHIKV IgM.

### Viral RNA extraction

Viral RNA was extracted automatically using the Maxwell 16 Viral Total Nucleic Acid Purification Kit and the Maxwell 16 Instrument (both Promega, Madison, WI, USA), from 300 μl of plasma, following the manufacturer’s instructions.

### Reverse transcription and DENV RNA detection

Reverse transcription was performed using the High-Capacity cDNA Reverse Transcription kit (ThermoFisher Scientific, São Paulo, Brazil) following the manufacturer’s instructions, except for the inclusion of a specific reverse DENV primer [13] instead of a random one. A protocol including an initial stage at 25 °C for 5 min followed by 37 °C for 2 h and terminating with a final denaturation at 85 °C for 5 min was applied.

DENV RNA was detected by TaqMan real-time PCR using forward (5´-GGACTAGAGGTTAGAGGAGACCCC-3′) and reverse primers (5´-GAGACAGCAGGATCTCTGGTC-3′), as well as the probe (FAM-AGCATATTGACGCTGGGA-MGB), which were capable of detecting all DENV serotypes and the majority of circulating genotypes in Brazil [13]. The amplification was performed in a final volume of 25 ul following a standard amplification protocol (initial activation at 50 °C for 5 min, denaturation at 95° for 10 min, and 40 cycles of 95 °C for 0:30 s and 60 °C for 1 min). As a positive control, a titrated viral stock of DENV-1 was used with a quantity of 10^7^ PFU/mL obtained after titration of the strain obtained from a male patient with Dengue hemorrhagic fever in VERO E7 cells. The sensitivity of the applied test was evaluated in-house using a serial dilution of the DENV-1 fragment cloned into the TOPO TA Cloning Vector (ThermoFisher Scientific, São Paulo, Brazil), which was obtained after cultivation of the virus from a sample taken from a patient with dengue hemorrhagic fever and a high viral load. The sensitivity of the test was 7 copies/DENV RNA per reaction.

### Statistical analysis

The online tool http://www.winepi.net [11] was used to calculate the prevalence of anti-DENV IgM and DENV RNA found in our study and the estimated true prevalence considering the total number of blood donations obtained for the studied period (28,238 blood donors). To do this, we took into account a specificity of 98% and a sensitivity of 100% for anti-DENV IgM tests, and perfect specificity and sensitivity for DENV RNA tests. When some DENV markers were absent in the studied population, the maximum possible prevalence for the overall population was calculated, considering the upper limit of the 95% CI.

## Results

Of the 475 blood samples collected, 315 (65.8%) were obtained from male blood donors (36.5 ± 10.8 years old) and 160 (34.2%) belonged to female (32.4 ± 10.2 years old) blood donors (Table 1). Considering the information in the Epidemiological Bulletin, a higher number of probable DENV cases occurred between December 2015 and May 2016 (Fig 1), peaking in February-March, which coincides with the rainy period of the study region.

**Table 1:** Studied population and DENV marker prevalence.

Gender	Male	Female	Total	Estimated true prevalence (CI: 95%)**
**n**	315 (65.8%)	160 (34.2%)	475 (100%)	**------**
**Age mean (±SD)**	36.5 (±10.8)	32.4 (±10.2)	35.1 (±10.7)	**------**
**Total IgM + BD**	24 (7.62%)	8 (5.00%)	32 (6.74%)*	4.83% (2.92 to 6.75%)
**IgM+/IgG+ BD**	14 (4.44%)	6 (3.75%)	20 (4.21%)*	2.26% (0.93 to 3.58%)
**Only IgM+ BD**	10 (3.17%)	2 (1.25%)	12 (2.53%)*	0.54% (0.00 to 1.19%)
**DENV NS1**	0 (0.00%)	0 (0.00%)	0 (0.00%)*	0.00% (0.00 to 0.63%)
**DENV RNA**	0 (0.00%)	0 (0.00%)	0 (0.00%)*	0.00% (0.00 to 0.63%)

SD: Standard deviation; BD: blood donors; CI: confidence interval;

*Apparent prevalence observed in this study;

**estimated true prevalence considering 28,238 blood donors.

Serological detection of anti-DENV IgM demonstrated a positive result in 6.75% (n = 32/475) of the donors (CI: 95%; 4.50 to 8.97%) (Table 1). The statistical analysis determined an estimated true prevalence of 4.83% (CI: 95%; 2.92% to 6.75%), considering the total number of individuals who donated blood in the studied period (28,238 blood donors). Six samples (n = 6/475. 1.26%) showed a result in the borderline zone for anti-DENV IgM (CI: 95%; 0.27% to 2.26%). We detected the highest peaks of prevalence of anti-DENV IgM in December (n = 8/82, 9.8%) 2015 and March (n = 7/81, 8.6%) and May (6/80, 7.5%) 2016. We believe that this reflects the initial stages of the outbreak (December 2015), while subsequent peaks might be related to intense proliferation of mosquito vectors and, therefore, a higher incidence of DENV infection. It is important to note that during the study we performed a search in the epidemiological bulletin for other arboviruses which may have co-circulated during the study period. There were 777 reported cases of suspected ZIKV infection and 442 suspected cases of CHIKV, which were very low compared with DENV (17,195 suspected DENV cases). Therefore, the detected prevalence of anti-DENV IgM indicating recent infection was in accordance with the predominant circulation of DENV when our study was performed. To rule out cross-reactivity, anti-DENV IgM-positive samples were also tested for anti-CHIKV IgM and anti-ZIKV IgM by MAC-ELISA. The results demonstrated no serological reactivity for anti-CHIKV IgM. On the other hand, 1 of 32 samples positive for DENV were positive for anti-ZIKV IgM and one was equivocal, but both samples presented titers much lower (~2 to 6 times) than that of anti-DENV IgM (data not shown).

The anti-DENV IgM-positive samples were additionally tested for anti-DENV IgG. Of the 32 that tested positive for anti-DENV IgM, 20 were also positive for anti-DENV IgG. Thus, samples that were positive for both anti-DENV IgM and IgG corresponded to 4.21% (n = 20/475) (CI: 95%; 2.42 to 6.00%) (Table 1). The simultaneous presence of anti-DENV IgM and IgG can occur in the late stage of primary DENV infection or in early secondary infection. Twelve samples (2.51%; n = 12/475) were positive only for anti-DENV IgM (CI: 95%; 1.13 to 3.93%) (Table 1), indicating the recent presence of infection.

No sample had a positive result for the DENV antigen NS1. The TaqMan real-time PCR, which detects all DENV serotypes, demonstrated negative results for DENV RNA in all of the tested samples. Considering the total number of blood donors in the studied period (28,238), the statistical analysis performed demonstrated that the maximum possible prevalence that we could expect for the NS1 and DENV RNA was 0.63% (0.00 to 0.63%; CI: 95%) (Table 1). Finally, none of the patients transfused with blood components that were positive for anti-DENV IgM developed symptoms consistent with arboviral infection (fever, rash, myalgia, arthralgia, or retro-orbital pain).

## Discussion

Dengue fever, the most prevalent arboviral disease in Brazil, represents a major public health problem because no governmental measures are directed at controlling the frequent outbreaks in the country [14]. DENV is of great consequence in the area of transfusion because the virus can be transmitted in blood and cause hemorrhagic outcomes in the recipients of blood components [15].

In early 2016, the region of the Federal District of Brazil experienced an extensive DENV outbreak, leading to a high number of notified cases as reported by SINAN (17,718 cases year-round with a peak season of January to May). This raises important issues regarding the safety of transfusions performed by hemotherapy services in this geographic location of Brazil. The detection of a 6.74% prevalence of anti-DENV IgM in blood donors demonstrated that recent infection had occurred [16]. This finding combined with the fact that at the blood donation interview, the donors denied any symptoms of acute infection in the past 6 months reveals that there was a significant percentage of asymptomatic DENV infections in donors who donated to the blood center during this period. One may question the specificity of the detected anti-DENV IgM due to the significant cross-reactivity between the different flaviviruses. From the epidemiological bulletin for 2016, we observed that DENV was the predominant circulating arbovirus during our study. Further, the comparison performed between the ZIKV and DENV IgM titers with MAC-ELISA demonstrated titers from 2 to 6 times higher for DENV, which is indicative of acute DENV infection rather than ZIKV, although a test with higher sensitivity for evaluation of antibody specificity, such as the Plaque Reduction Neutralization Test (PRNT), is needed. None of the samples positive for anti-DENV IgM were reactive for anti-CHIKV IgM.

A high prevalence of DENV asymptomatic viremia has been shown in Brazilian blood donors during outbreaks in Rio de Janeiro (0.51%) and Recife (0.80%) [3], which can lead to an elevated prevalence of anti-DENV IgM during DENV outbreaks in asymptomatic donors. This is in accordance with our results demonstrating a high proportion of recent DENV infection in the Federal District. We do not believe that the negative result for detection of DENV viremia was related to low sensitivity of the applied real-time PCR test. In an in-house evaluation of the test’s sensitivity, we observed a high detection limit, i.e., ~7 copies/mL. Moreover, during the asymptomatic viremic phase, DENV loads can exceed 10^6^ copies/mL [17], which is indicative that our real-time PCR test would not have missed viremic infections.

In the majority of cases (62.5%), anti-DENV IgM was detected simultaneously with anti-DENV IgG. Such a finding probably means that the acute infection was in its final phase after viremia [16]. In line with this, we obtained negative results for both NS1 and DENV RNA in the tested blood donors. Since all our samples were DENV RNA negative, the negative NS1 antigen result was expected, as NS1 testing shows even lower sensitivity than PCR (sensitivity provided by the manufacturer 91%, CI95%: 85.5%-94.8%). Therefore, the negative result obtained by NS1 was in complete agreement with the results obtained by DENV real-time PCR. On the other hand, the simultaneous presence of anti-DENV IgM and anti-DENV IgG may also occur in an early secondary DENV infection [16] with the presence of DENV RNA. However, our data do not support this explanation because we could not detect DENV RNA in the tested samples.

The lack of detection of DENV RNA may occur for one of the following possible reasons: (i) testing of a relatively low number of randomly selected tested samples, which may miss viremic donations and (ii) the very short-lived viremia of DENV during acute infection. In support of the lack of detection due transient viremia, other studies in Brazil, performed in the Amazonas and São Paulo State during a DENV outbreak, did not succeed at detecting DENV in any of the 205 samples tested despite the sensitivity and specificity of the applied tests [18]. In Brazil, the prevalence of DENV RNA during DENV outbreaks varies between 0.4% and 0.8% [3,19], depending on the epidemic year. The upper limit of the 95% CI demonstrated a maximum prevalence of DENV RNA of 0.63% (CI: 95%) if we had tested all donated blood for DENV RNA. Similarly, studies from other geographic locations demonstrated a prevalence of DENV RNA between 0.0 (China, Australia) [20–22] and 0.07% (Puerto Rico) [23]. In all cases, the detected prevalence of DENV RNA was lower than those of studies performed in Brazil [3] and Saudi Arabia (5.5%) [24]. Such highly variable results probably depend on the presence of a DENV outbreak, geographic localization, and whether the method of DENV RNA detection can detect all DENV serotypes. We believe that in our case, DENV can pose a threat to the blood supply in the Federal District due to the fact that none of the anti-DENV IgM positive blood donors experienced any symptoms, and if they had donated during the viremic stage of the infection, they would probably have caused viremic blood components to be transfused.

Seroepidemiologic studies represent an important methodology for understanding the real burden of disease in the studied population. Such strategies may include tests for anti-DENV IgM, which reflect the number of acute infections and the circulation of DENV during outbreaks. In our study we detected a high prevalence of anti-DENV IgM (6.74%), which is similar to the seroprevalence obtained in Rio de Janeiro city in Brazil (8.8%) [25] and Saudi Arabia (5.5%) [24]. However, higher indexes of anti-DENV IgM were observed in India (13.5%) [26]. In this regard, surveys among asymptomatic donors in China during the 2014 outbreak reported that the anti-DENV IgM prevalence rate was 2.4% [22]. During the next outbreak (2015), the prevalence of anti-DENV IgM declined to 0.4%. This decrease may have been a result of actions of the Chinese Center for Disease Control (CDC), which improved control and prevention strategies for DENV [20], but may also have been a result of lower circulation of DENV (non-epidemic year) or circulation of a different serotype. In contrast, a much lower seroprevalence was detected in Singapore (2.83%) and Australia (0.22%), regions in which DENV is endemic; however, they represent developed countries with a possible implementation of control measures to curb DENV outbreaks. Probably, the lower seroprevalence of anti-DENV IgM in the above-mentioned geographic localities are due to the stringent donor selection process for blood donation in both areas [21,27].

## Conclusion

In conclusion, this study, which examined the prevalence of DENV IgM/anti-DENV IgG and DENV RNA in blood donors from Central-West Brazil during an outbreak in 2016, demonstrated that 6.75% of tested blood donors had experienced a recent asymptomatic DENV infection, as defined by a DENV IgM positive result, which shows that a significant portion of the population could be asymptomatic DENV carriers. Although we detected neither DENV RNA nor NS1 antigen, the observed high prevalence of anti-DENV IgM in healthy donors who reported no symptoms at the time of donation indicates possible participation of this virus in TT-DENV in the examined region.
